# Identification of a VapA virulence factor functional homolog in *Rhodococcus equi* isolates housing the pVAPB plasmid

**DOI:** 10.1371/journal.pone.0204475

**Published:** 2018-10-04

**Authors:** Jennifer M. Willingham-Lane, Garry B. Coulson, Mary K. Hondalus

**Affiliations:** Department of Infectious Disease, University of Georgia, Athens, Georgia, United States of America; University of Parma, ITALY

## Abstract

*Rhodococcus equi* is a facultative intracellular bacterium of macrophages and is an important pathogen of animals and immunocompromised people wherein disease results in abcessation of the lungs and other sites. Prior work has shown that the presence of the major virulence determinant, VapA, encoded on the pVAPA-type plasmid, disrupts normal phagosome development and is essential for bacterial replication within macrophages. pVAPA- type plasmids are typical of *R*. *equi* strains derived from foals while strains from pigs carry plasmids of the pVAPB-type, lacking *vapA*, and those from humans harbor various types of plasmids including pVAPA and pVAPB. Through the creation and analysis of a series of gene deletion mutants, we found that *vapK1* or *vapK2* is required for optimal intracellular replication of an *R*. *equi* isolate carrying a pVAPB plasmid type. Complementation analysis of a Δ*vapA R*. *equi* strain with *vapK1* or *vapK2* showed the VapK proteins of the pVAPB-type plasmid could restore replication capacity to the macrophage growth-attenuated *ΔvapA* strain. Additionally, in contrast to the intracellular growth capabilities displayed by an equine *R*. *equi* transconjugant strain carrying a pVAPB-type plasmid, a transconjugant strain carrying a pVAPB-type plasmid deleted of *vapK1* and *vapK2* proved incapable of replication in equine macrophages. Cumulatively, these data indicate that VapK1 and K2 are functionally equivalent to VapA.

## Introduction

The Gram-positive aerobic bacterium, *Rhodococcus equi*, is found in the soil worldwide and in the fecal matter of herbivores [1, 2]. In addition to being an environmental saprophyte, *R*. *equi* is a facultative intracellular pathogen of macrophages, capable of causing disease in a variety of susceptible hosts. The most well-studied disease presentation is in foals, but other hosts include pigs, cattle, and humans. In foals and people, bacterial exposure is generally through inhalation and disease results in pneumonia with the formation of pyogranulomatous lesions within the lungs [2–4]. Disease in swine and cattle typically presents as submandibular lymphadenitis and abscessation of the respiratory lymph nodes, respectively [5–8]. All *R*. *equi* isolates from diseased foals and most from affected swine and humans carry a large circular virulence-associated plasmid, whereas, cattle *R*. *equi* isolates possess a linear plasmid [9–11]. The *R*. *equi* isolates obtained from equine, swine, and cattle carry distinct plasmid types referred to as pVAPA, pVAPB, and pVAPN respectively whereas, *R*. *equi* strains infecting humans may carry any of these plasmid types. Located on all of these plasmids is a pathogenicity island (PAI) acquired from a historical horizontal gene transfer event [9]. Though the genetic composition the PAI regions of these plasmid types vary, all contain a novel family of genes known as the *v**irulence* *a**ssociated* *p**roteins* or *vap* family [9, 11].

*R*. *equi* isolates from diseased foals have been characterized more thoroughly than isolates from other hosts. It is well established that plasmid possession by the equine isolate is essential for virulence *in vivo* because it enables the bacteria to replicate in host macrophages, a fundamental feature of disease pathogenesis [12, 13]. Within the equine pVAPA–type plasmid PAI, there are 6 full length *vap* genes (*vapA*, -*C*, -*D*, -*E*, -*G*, and -*H*) along with 3 *vap* pseudogenes (vap*F*, -*I*, and -*X*) [9, 11]. Of these six full length *vap* genes, *vapA* has been shown to be a key virulence factor and encodes the surface-localized VapA protein that is essential for bacterial intramacrophage replication as established by the inability of a *vapA* deletion mutant to replicate in host macrophages. [14]. In addition to *vapA*, two regulatory genes, *virR* and *virS* which are a part of the five gene *virR* operon, are also required for intramacrophage growth and function by regulating *vapA* expression along with that of ~ 18% of chromosomal genes [15]. While the exact function of VapA remains elusive, the protein has recently been observed to localize to the phagosome membrane during macrophage infection [16] and its presence is associated with disruption of phagosome maturation and acidification thereby creating an intracellular compartment favorable for bacterial replication [17–19].

While equine isolates possess the essential virulence component *vapA*, neither the pVAPB nor pVAPN plasmids typical of swine and bovine isolates respectively, contain this gene. Interestingly, the specific *vap* genes present are distinct among the three PAI regions while the *virR* operon is largely conserved in all three plasmid types. The pVAPB-type plasmid includes 6 full length *vap* genes (*vapB*, -*J*, -*K1*, -*K2*, -*L*, and -*M*) while pVAPN1571 carries four full length *vap* genes (*vapN*, -*O*, -*P*, and -*Q*) along with two *vap* pseudogenes (*vapR* and *vapS*) [9, 10]. Recently, Vazquez-Boland and colleagues showed that the pVAPN-type plasmid equips the organism for replication in macrophages and identified the requirement of the PAI-encoded *vapN* gene for this trait [10]. Deletion of *vapN* yields a strain unable to replicate in murine macrophages, a phenotype resembling that of a pVAPA *vapA* deletion mutant.

We recently demonstrated possession of the pVAPB-type plasmid, typical of most swine isolates and commonly found in *R*. *equi* strains cultured from people, enables the bacterium to replicate in murine, equine, and swine macrophages [20]. Since the pVAPB-type plasmid does not contain the *vapA* or *vapN* genes, the objectives of this study were to determine the component/s of the pVAPB-type plasmid that allow/s for replication within macrophages and assess whether the identified component/s is/are functionally equivalent to the *vapA* gene encoded on the equine pVAPA-type plasmid.

## Materials and methods

### Ethics statement

Macrophage precursor cells were collected from Balb/c mice and equine alveolar macrophages were collected from two individual horses using protocols approved by UGA Institutional Animal Care and Use Committee (IACUC). Approval numbers A2017 01-004-Y2-A4 and A2016 05-014-Y1-A respectively.

### Bacterial strains, culture media and growth conditions

Bacterial strains used in this work are listed in S2 Table. *R*. *equi* strains were grown at either 30°C or 37°C (shaking 200rpm) in Brain Heart Infusion Broth (BHI) or minimal acetate medium (MM-Ac) broth. MM-Ac contained K_2_HPO_4_ (4.65g/L), NaH_2_PO_4_.H_2_O (1.5g/L), sodium acetate (10g/L), NH_4_Cl (3g/L), MgSO_4_.7H_2_O (1g/L), thiamine (40mg/L), and Vishniac stock solution (1mL/L). The Vishniac stock solution was prepared as follows: EDTA (10g/L) and ZnSO_4_.7H_2_O were dissolved in 900mLs dH2O and the pH was adjusted to 8.0 with 2M KOH to allow for dissolution of the salts [21]. Then CaCl_2_.2H_2_O (1.47g/L), MnCl_2_.7H_2_0 (1.0g/L), FeSO_4_.7H_2_O (1.0g/L), (NH_4_)_6_Mo_7_O_24_.4H_2_0 (0.22g/L), CuSO_4_.5H_2_0 (0.32g/L), and CoCl_2_.6H_2_O (0.32g/L) were added and the pH adjusted to 4.0 and the final volume adjusted to 1L. For agar plating, granulated agar (15g/L) was added. For selection during mutagenesis studies, 5-Fluorocytosine (Sigma-Aldrich) stock solution (10mg/mL) was prepared in distilled H_2_O, dissolved by heating to 50°C, and then filter sterilized and added to autoclaved minimal media (100ug/mL) [21]. When necessary, antibiotics are added at the following concentrations: apramycin, 80μg/mL; hygromycin, 180μg/mL; zeocin 50μg/mL.

### Electroporation of *R*. *equi*

To create electrocompetent *R*. *equi*, the bacteria was grown in BHI broth, with antibiotic as appropriate, to an OD_600nm_ of ~0.8–1.0. Bacteria were pelleted and washed twice with equal volumes of cold sterile water. After the final centrifugation, the pellet was resuspended in a solution of cold 10% glycerol in water at 1:20th of the original culture volume. Aliquots of 400μL were made and stored at -80°C until use. For electroporation, thawed cells were mixed with ~500ng of plasmid DNA and placed in a chilled 0.2cm electroporation cuvette. Electroporation was performed using a Gene Pulser Xcell system (Bio-Rad) set at 2.5kV, 25μF, 1000Ω, and single pulse. Immediately after electroporation, 1mL of BHI supplemented with 0.5M sucrose was added to the cells which were then incubated at 30°C for 60 minutes and plated on BHI agar.

### Mutant construction

Primers used in the construction and analysis of mutant strains are listed in S3 Table.

#### 33705ΔPAI deletion mutant

To construct a PAI-deletion mutant in the pVAPB-type *R*. *equi* strain 33705, primers PAI-up (DraIII)-F and PAI-up (SpeI)-R were used to amplify sequence upstream of the PAI region yielding a 962bp amplicon which was sequentially digested with DraIII and SpeI then ligated into the pSelAct suicide vector [21] digested in the same manner creating pJWL1.1. The 935bp downstream region of the PAI was amplified using primers PAI-down (SpeI)-F and PAI-down (EcoRI)-R then digested with EcoR1 and SpeI and ligated to the similarly digested pJWL1.1 construct, producing pJWL1.2. In order to obtain the hygromycin resistance cassette, vector pMV261.hyg was digested with DraI/Hpa1 which generated a 1.3kb fragment containing the hygromycin resistance cassette which was then gel purified and treated with Klenow (New England Biologicals) to create blunt ends. The cassette was then ligated to pJWL1.2 which had been previously digested with SpeI and similarly Klenow treated thereby placing the cassette between the upstream and downstream regions of the PAI region, yielding the suicide vector pJWL1.0hyg. Allelic exchange was achieved utilizing the method described by van der Geize [21], which uses the products of the *codA*::*upp* genes as a counterselection mechanism. Briefly, 500ng of pJWL1.0hyg was electroporated into *R*. *equi* strain 33705 and transformants were selected on BHI agar supplemented with hygromycin and apramycin. The transformants were confirmed to be sensitive to 5-Fluorocytosine (5-FC) by plating on MM-Ace Agar supplemented with 5-FC and were considered to have undergone a single homologous recombination event. In order to promote a secondary recombination, during which the vector backbone sequences (i.e. apramycin resistance cassette, *codA*::*upp* genes) were lost, single-cross recombinant strains were serially subcultured in BHI broth supplemented with hygromycin at 30°C. In order to recover putative PAI mutants, dilutions of the subculture were plated on MM-Ace agar supplemented with hygromycin and 5-FC. Putative PAI deletion mutant clones resistant to both hygromycin and 5-FC were screened for sensitivity to apramycin. Subsequently the genotypes of the putative mutant clone was confirmed by PCR analysis.

#### 33705Δ*vapB* deletion mutant

Primer pairs vapB-up-F (DraIII) and vapB-up-R (SpeI) were used for amplification of a 848 bp fragment of upstream *vapB* sequence which was subsequently digested with DraIII and SpeI and ligated with the similarly digested pSelAct vector creating pGBC26. Primer pairs vapB-down-F (SpeI) and vapB-down-R (XmaI) were used to amplify the 770 bp downstream flank which was then sequentially digested with SpeI and XmaI and ligated with pGBC26 digested with SpeI/XmaI resulting in pGB26.1. pGB26.1was digested with SpeI and a hygromycin cassette was inserted between the upstream and downstream *vapB* sequences in the same manner as the PAI deletion mutant. The allelic exchange procedure was then followed as described above for creation of the PAI deletion mutant.

#### 33705Δ*vapK1-vapMΔvapB* deletion mutant

The suicide vector pJWL2.0 was used to delete a 4.7 kb region ranging from *vapK1* to *vapM* on the 33705Δ*vapB* background. To begin, a 938 bp region upstream of *vapK1* was amplified using primer pair vapK1-up(DraIII)-F and vapK1-up(SpeI)-R. The amplicon was digested with SpeI/DraIII and ligated into similarly digested pSelAct vector creating pJWL2.1. Next a 747 bp region downstream of *vapM* was amplified using primer pairs vapM-down(SpeI)-F and vapM-down(PciI)-R. Both the downstream amplicon and pJWL2.1 were digested with PciI/SpeI and ligated together to produce pJWL2.0 which was ultimately used to transform electrocompetent *R*. *equi* strain 33705Δ*vapB*. Allelic exchange was achieved by serial subculturing in the presence of hygromycin at 30ºC and selection of the double-cross was done as described above for creation of the PAI mutant.

#### 33705Δ*vapK1* deletion mutant

The deletion of *vapK1* on the wild type 33705 pVAPB-type plasmid background was done using the suicide vector pJWL4.0. Cloning was initiated by first amplifying the downstream region of *vapK1* using primer pairs vapK1-down (SpeI)-F and vapK1-down (XmaI)-R resulting in a PCR product of 1.3 kb in size that was digested with SpeI/XmaI and ligated with the similarly digested pJWL2.1, creating pJWL4.1. Additionally, the pEM7/Zeo plasmid (ThermoScientific) was digested with XbaI and EcoRV resulting in the isolation of a zeocin resistance cassette under the EM7 bacterial promoter (477 bp). For positive selection of clones, a zeocin resistance cassette was inserted by digesting pJWL4.1 with SpeI and following Klenow treatment of both the pJWL4.1 digested vector and zeocin resistance cassette the two components were ligated together producing pJWL4.0. pJWL4.0 was used to transform wild type *R*. *equi* 33705 and transformants were selected on BHI agar with zeocin and apramycin. Subsequently allelic exchange was performed as described earlier for construction of the PAI mutant except that subculturing was done in BHI supplemented with zeocin.

#### 33705Δ*vapB*Δ*vapK2* and 33705Δ*vapK1*Δ*vapK2* deletion mutants

An in-frame unmarked deletion of *vapK2* was created using the suicide vector pJWL5.0. A 967 bp upstream sequence of vapK2 was amplified using primer pairs K2-up-F and K2-up-R both of which contained 5’-phosphates. Next the pSelAct vector was digested with SmaI, treated with Antarctic Phosphatase (New England Biologicals), and then ligated to the amplified and gel purified upstream *vapK2* PCR product creating pJWL5.1. Restriction digestion was used to confirm proper *vapK2* upstream insert orientation. The downstream region of *vapK2* was amplified using primer pairs containing 5’-phosphates (vapK2-down-F and vapK2-down-R) producing a 1.1 kb PCR product. Next, pJWL5.1 was digested with EcoR1 then Klenow and phosphatase treated, and then ligated with the downstream *vapK2* amplicon yielding pJWL5.0. Proper orientation of the *vapK2* downstream fragment in pJWL5.0 was confirmed by restriction digestion. pJWL5.0 was used to transform *R*. *equi* strains 33705*ΔvapB* and 33705*ΔvapK1*. Allelic exchange for 33705*ΔvapBΔvapK2* and 33705*ΔvapK1ΔvapK2* was achieved by serial subculturing in the presence of hygromycin or zeocin, respectively, and putative vapK2 deletion mutant clones that were resistant to both hygromycin or zeocin and 5-FC were screened for sensitivity to apramycin.

### Creation of complementing vectors

The episomal multi-copy pMV261.hyg vector [22] was utilized in a complementation analysis of strains 33705Δ*vapK1*Δ*vapK2* and 103SΔ*vapA* wherein *vapK1*, and *vapK*2 each were expressed individually from the *Mycobacterium spp*. *hsp60* promoter. *vapK1* was amplified using the 5’ phosphorylated primer pair vapK complement-F and vapK1 complement-R resulting in a 756 bp amplicon. The vapK complement-F and vapK2 complement-R primer pair with 5’-phosphates was utilized to amplify *vapK2*, producing a 834 bp PCR product. The DNA template used to amplify *vapK1* and *vapK2* was from 33705Δ*vapB*Δ*vapK2* and 33705Δ*vapK1*, respectively. pMV261.hyg was digested with MscI whose recognition site is located directly after the start codon for the *Mycobacterium spp*. *hsp60* gene. Following restriction digestion and phosphatase treatment, the vector was individually ligated to the *vapK1* or *vapK2* amplicons creating *pMV261*.*hyg*.*vapK1* and *pMV261*.*hyg*.*vapK2* respectively. Each complementing vector was electroporated into 33705Δ*vapK1*Δ*vapK2* and 103SΔ*vapA* in the manner previously described.

### Creation of transconjugant isolates

Mating was performed as described by *Tripathi et al* [23]. The virulence plasmid cured 103S^P-^ derivative was chromosomally marked with the gene *[aac(3)-IV]* providing apramycin resistance via electroporation with pSET152 (designated Apr^R^ or A) [23]. For virulence plasmid transfer, equal numbers of chromosomally marked recipient (103S^P-^/A) and zeocin marked donor 33705Δ*vapK1*Δ*vapK2* strain was used. The donor and recipient strains were grown overnight at 37°C in BHI broth supplemented with the appropriate antibiotic. The next day the OD_600nm_ was adjusted to 1.0 (~2x10^8^ CFU/mL). Approximately 10^7^ CFU of both donor and recipient bacteria were mixed in a small volume (5–10μL), and spotted on BHI agar and the plates incubated for 72 hours at 30°C. Afterwards, the cell mixture was scraped from the plates and resuspended in 1mL PBS. Serial dilutions (up to 10^−7^) of the resuspended cells were plated on agar containing the appropriate antibiotics for selection of transconjugants as well as recipients and donors. Putative transconjugant colonies were screened for the presence of the transferred virulence plasmid using PCR analysis to confirm the presence of the replication/partitioning (primers REVP1/c; S3 Table), conjugation (primers REVP6/c; S3 Table), unknown function (primers REtrbA1/c;S3 Table), and pathogenicity (primers vapA-F, vapA-R or vapB-int-F, vapB-int-R; S3 Table) regions.

### Bone marrow-derived macrophages

To obtain macrophage precursors, the femurs and tibias of female BALB/c mice were flushed into a 50mL conical tube with cold cation-free Phosphate-Buffered Saline (PBS) supplemented with penicillin G (100U/mL)-streptomycin (100ug/mL) (PSG). The cells were centrifuged at 1100rpm for 10min at 4°C. The cell pellet was resuspended in complete media consisting of DMEM containing 10% fetal bovine serum (FBS), 10% cell culture supernatant from Colony Stimulating Factor 1 (CSF-1) producing L929 cells and 2mM glutamine, and washed by centrifugation at 1100rpm for 10 minutes. The cells were resuspended in 24mL per mouse of complete media. The precursor cells were plated in 6-well non-tissue culture treated plates (4mLs per well) and incubated at 37°C with 5% CO_2_. On the third day, 4mL of complete media was added to each well. On day 5, the media was removed and replaced with complete media without antibiotic, then on day 6, the media was aspirated and each well washed with 4mL PBS to remove any non-adherent/dead cells. Then 4mLs of cold cation-free PBS was added and the plates refrigerated at 4°C for 15 minutes. Afterwards, the cells were collected, quantified, and seeded into tissue-cultured treated 24-well plates at a concentration of 2x10^5^ per well.

### Equine alveolar macrophages

To acquire alveolar macrophages, a bronchoalveolar lavage (BAL) was performed on adult horses sedated with xylazine hydrochloride (05.mg/kg, IV) and butorphanol tartrate (0.1 mg/kg, IV). A sterile BAL catheter was passed via the nasal cavity and wedged within a bronchus. Four aliquots of 60mL (~240 mL total) of sterile physiologic saline (0.9% NaCl) solution was infused into the horse’s lungs and aspirated immediately. Once collected, the BAL fluid was centrifuged at 1100 rpm for 10 minutes at 4°C and the pellet resuspended in 50mL PBS followed by another similar centrifugation. This washing step was repeated two additional times. The final cell pellet was resuspended in Minimum Essential Medium (MEM) Alpha supplemented with 10% Donor Horse Serum (DHS) (ThermoScientific), 2mM glutamine and PSG. Upon quantification, 4 x 10^5^ cells were pipetted into each well of a 24-well tissue culture plate, wherein each well contained a 13mm glass coverslip. The plates were incubated at 37°C with 5% CO_2_ for 4 hours to allow for macrophage adherence. After 4 hours, the wells were washed 3 times with MEMα to remove non-adherent cells, then the media was replaced with antibiotic free MEMα+10% DHS+ 2mM glutamine and the cells incubated overnight at 37°C with 5% CO_2_.

### Bacterial intracellular growth assay

Overnight bacterial broth cultures were grown to an optical density_600nm_ (OD_600nm_) of 1.0 (~2.0x10^8^ CFU/mL) and washed once with PBS and resuspended to the original culture volume in PBS. Macrophage monolayers (BMDM or equine macrophages) were washed once with warm DMEM. The medium was replaced with DMEM supplemented with 10% FBS and 2mM glutamine (equine), or 10% FBS, 10% CSF-1, and 2mM glutamine (BMDMs). Bacteria were added at a multiplicity of infection (MOI) of 10 bacteria per macrophage. Following 60 min of incubation at 37°C, the monolayers were washed 3 times with DMEM to remove any unbound bacteria and the appropriate media containing 20μg/mL of amikacin sulfate was added to the monolayers in order to prevent extracellular bacterial replication. At various times post-infection, the macrophage monolayers were washed repeatedly and lysed by the addition of 500μL of sterile water, and the lysate was collected and plated onto BHI agar. The number of colony forming units (CFU) associated with the macrophage lysate was determined after a 72 h incubation at 37°C (BMDMs). Alternatively, the monolayers were fixed with cold methanol and the associated bacteria were stained with polyclonal rabbit anti-*R*.*equi* antibody followed by a FITC-labeled goat-anti-rabbit secondary antibody allowing for the enumeration of the bacteria under fluorescent microscopy.

### Fluorescent staining of *R*. *equi* infected macrophages

Infected macrophage monolayers were fixed onto glass coverslips with 100% cold methanol for 30 minutes at 4°C. The monolayers were washed once with PBS then primary polyclonal rabbit anti-*R*. *equi* antibody, diluted 1:1000 in PBS containing 5% Normal Goat Serum (NGS), was added to the fixed monolayers and incubated for 60 minutes at room temperature (RT). Following washing with PBS containing 5% NGS, goat anti-rabbit antibody conjugated with Alexa Fluor 488 (diluted 1:1000 in PBS with 5% NGS) was added and the monolayers incubated for 60 min at RT. Following washing with PBS containing 5% NGS, the coverslips were mounted onto microscope slides using ProLong Gold containing DAPI stain (Invitrogen).

### RNA isolation

Total RNA was isolated from 5mL bacterial culture grown to mid-logarithmic stage (O.D. of 0.8–1.0) after harvesting by centrifugation at 4,000xg for 10 minutes. Alternatively, total RNA was isolated from intracellular *R*. *equi* grown in *in vitro* cultured macrophages as previously described [15]. *R*. *equi* RNA was isolated from macrophages following phagocytosis of the pathogen using a guanidine thiocyanate-based lysis buffer (4 M guanidine thiocyanate, 0.5% [wt/vol] sodium N-lauryl sarcosine, 25 mM sodium citrate, and 0.1 M-mercaptoethanol). *R*. *equi* infected macrophages were vortexed and passed through a needle to shear macrophage DNA and intracellular bacteria were recovered by centrifugation. In either case, the bacterial pellet was resuspended in RLT buffer supplied by Qiagen RNeasy mini kit and added to 0.1-mm-diameter silica beads (Bio-Spec). The bacterial samples were lysed by vortexing on high for 5 minutes. Total RNA was subsequently isolated using the Qiagen RNeasy mini kit according to the manufacturer’s instructions.

### RT-qPCR analysis

Total RNA in each sample was quantified by NanoDrop, and cDNA was synthesized using iScript Reverse Transcription Supermix for RT-qPCR (Bio-Rad) from ~1ug of RNA template following instructions provided by the manufacturer. 5uL of the cDNA reaction mixture was used as template for qRT-PCR analysis with iTaq Universal SYBR Green Supermix (Bio-Rad). Primer pairs specific for *vapL* (vapL-qRT-F/R), *vapM* (vapM-qRT-F/R) and *vapK* (vapK-qRT-F/R) were used to determine the expression of these genes relative to the housekeeping gene *gyrB* (gyr-qRT-F/R).

### Statistical analysis

Statistical analyses of bacterial growth assays were performed using the SigmaPlot statistical package (Systat Software, San Jose, CA). Normality and equality of variance of the data were assessed with use of the Shapiro-Wilk and Levene tests, respectively. Intracellular numbers of bacteria were compared by one-way or two-way repeated measures analysis of variance (ANOVA) from a compilation of three independent experiments. When appropriate, multiple pairwise comparisons were performed using the method of Holm-Sidak. Significance was set at a p-value of p<0.05.

## Results

### Deletion of the PAI region of a pVAPB-carrying *R*. *equi* isolate abolishes the capacity for intracellular replication

**Fig 1:**
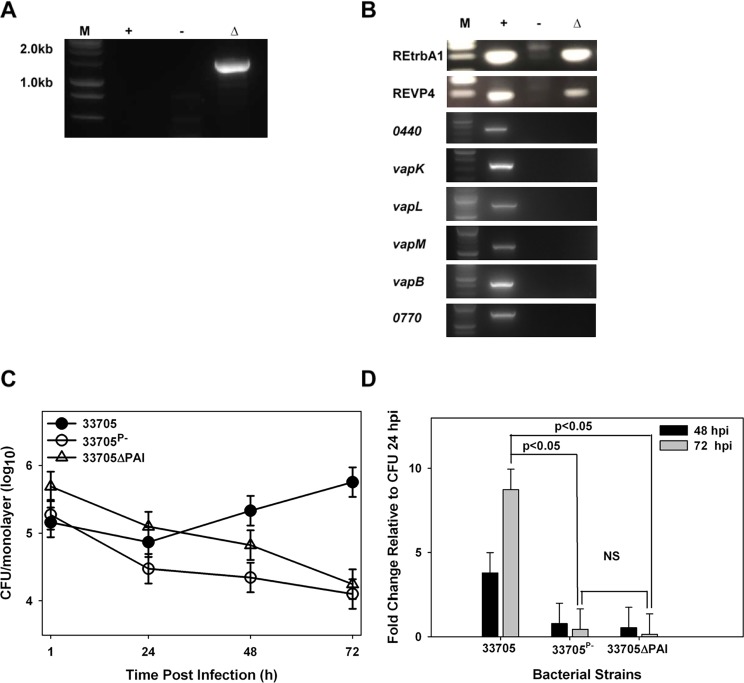
The PAI region of the *R*. *equi* pVAPB-type plasmid is required for intracellular growth in murine macrophages. PCR analysis of the pVAPB-type PAI deletion mutant using primer pairs to show the replacement of the PAI region with a hygromycin resistance cassette (A) and numerous primer pairs to confirm the deletion of this region in its entirety (*0440*; *vapK*; *vapL*; *vapM*; *vapB*; *0770*) with retention of the plasmid backbone (REtrbA1; REVP4) (B). Results obtained for the PAI mutant are shown in the right most lanes labeled Δ. The second and third lanes are the products of control PCR reactions using total genomic DNA from a wild type strain carrying a pVAPB-type plasmid or from a plasmid-cured strain as template, indicated by + and–symbols respectively. Standard molecular weight DNA markers (M) are in the left most lane. Intracellular growth was assessed in murine bone marrow derived macrophages (BMDM) infected with wild type strain 33705, plasmid-cured 33705^P-^, and 33705ΔPAI at an MOI of 10:1 and was followed over 72 h post infection (hpi) by lysis and plating of triplicate macrophage monolayers (C). Fold change in CFU of intracellular bacteria at 48 hpi and 72 hpi relative to 24 hpi (D). Statistical analysis was on a compilation of 3 individual experiments. Error bars represent the standard deviation from the mean. NS: not significant.

The backbone regions of the *R*. *equi* plasmids pVAPA and pVAPB are nearly identical. It has been shown that deletion of the PAI region of the pVAPA plasmid, housing *vapA*, yields an attenuated strain [9, 24]. Based on these data, we focused on the PAI of the pVAPB-type plasmid as the likely location for intracellular growth-essential genes. To assess whether the PAI region was critical for growth in macrophages, we constructed a PAI deletion mutant in the pVAPB-type carrying *R*. *equi* strain 33705 using targeted allelic replacement as described in the *Materials and Methods*. The genotype of the PAI mutant was verified by PCR analysis using primer pairs that annealed external to the deletion site (S3 Table and Fig 1A). As expected, a ~1.5 kb size band was amplified in the deletion mutant due to the presence of a hygromycin resistance cassette marking the site of the deletion. No amplicon was produced when the wild type plasmid was used as template because the PAI region is too large to amplify under the PCR conditions utilized. Furthermore, PCR analysis using primer pairs which annealed to various genes within the PAI region (*0440*; *vapK*; *vapL*; *vapM*; *vapB*; *0770*) was done to verify the loss of those genes in the mutant and primers annealing to the plasmid backbone region (REtrbA1; REVP4) confirmed its presence in the mutated plasmid (Fig 1B). Cumulatively, these PCR results demonstrate the successful deletion of the PAI in *R*. *equi* strain 33705 background and the retention of an intact plasmid backbone.

To determine if deletion of the PAI region of pVAPB had any effect on the intramacrophage replication ability of the strain, murine bone-marrow derived macrophages (BMDMs) were infected with the PAI deletion mutant and its growth phenotype was compared to that of wild type strain 33705 possessing its native pVAPB plasmid, as well as, its plasmid-cured derivative strain 33705^P-^. The murine macrophage is a well-established *in vitro* model system to analyze *R*. *equi* intracellular growth and results observed correlate with strain virulence [13, 14, 24]. Bacterial growth was followed using standard lysis and plating of *R*. *equi* infected macrophage monolayers and data from each experiment were expressed in two formats, first as the number of colony forming units (CFU) per macrophage monolayer over time (Fig 1C), and secondly as fold change in bacterial numbers relative to 24 hours post infection (hpi) (Fig 1D). As shown, the pVAPB-containing wild type isolate replicated ~10-fold over 72 hours relative to 24 hours post infection (hpi), whereas, the PAI deletion mutant failed to replicate and was as attenuated for intracellular growth as the strain lacking a virulence-associated plasmid. These data confirm, that as suspected, the PAI region of the pVAPB-type plasmid houses a component(s) required for intramacrophage replication.

### Deletion of *vapK1* through *vapM* results in the loss of intracellular growth capability of a pVAPB-carrying *R*. *equi* isolate

**Fig 2:**
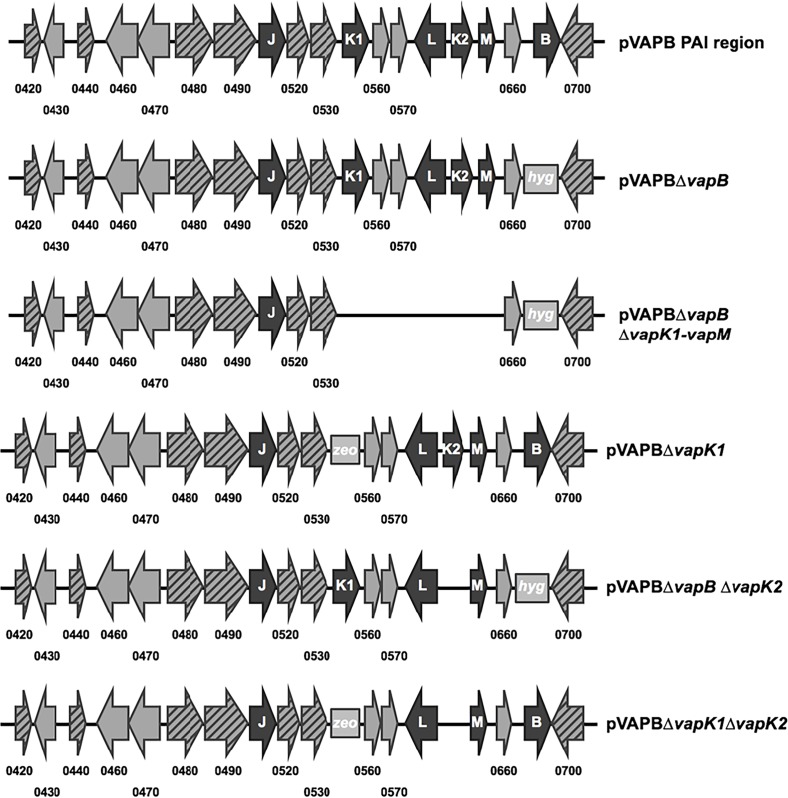
Schematic diagram showing the various pVAPB-type plasmid mutants constructed and analyzed. Regions of gene conservation among the pVAPB, pVAPA, and pVAPN-type plasmids are represented by grey arrows containing black dashes. Genes that are not highly conserved among the various plasmid types are shown in grey arrows and the pVAPB-type plasmid *vap* family members are indicated by black arrows. Deletion sites marked by an antibiotic cassette are indicated by square grey boxes containing the respective antibiotics abbreviation (*hyg*:hygromycin; *zeo*:zeocin).

Since the data show the necessity for the PAI region of the pVAPB-type plasmid for growth in macrophages, a series of *R*. *equi* mutant strains, deleted for various parts of the PAI region, were constructed to identify the gene/s required for intracellular replication (Fig 2) and the effects of the specific deletion on intramacrophage fitness was analyzed. Since *vapA* is known to be essential for intracellular replication of *R*. *equi* isolates carrying the pVAPA-type plasmid [14], we speculated that the relevant gene(s) within the PAI region of the pVAPB-type plasmid would likewise be a *vap* family member. Because VapB is the protein with the highest degree of sequence identity (75%) to VapA, and it had been proposed that it was likely functionally equivalent to VapA, we first created a mutant that was deleted for *vapB* [9]. The deletion of *vapB* was confirmed by PCR analysis using primer pairs (S3 Table) annealing internal to the gene that produced a 411 bp band when wild type 33705 DNA was used as template (S1A Fig lane 2). This band was absent in the mutant strain (S1A Fig lane 4). Additionally, PCR analysis using primer pairs (S3 Table) that annealed just outside of *vapB* showed that the 650 bp amplicon produced by wild type template was replaced by a 1.5 kb band indicative of the presence of the hygromycin resistance cassette marking the deletion site (S1B Fig). Surprisingly, deletion of *vapB* had no effect on the strain’s ability to replicate within murine macrophages, wherein its growth was identical to the parent strain (S1C and S1D Fig). Because of its wild type phenotype and being marked by an antibiotic resistance cassette, most of the future deletions were constructed on the 33705Δ*vapB* background wherein antibiotic pressure could be used to facilitate virulence plasmid retention during the allelic replacement procedure.

We next created a mutant strain on the *ΔvapB* background that had an additional 4.7 kb deletion in the PAI, specifically a region ranging from *vapK1* through *vapM*, resulting in the removal of 4 more *vap* genes (*vaps K1; L; K2; M*) and 2 genes of unknown function (*0560*;*0570*). Fig 2 shows a schematic representation of this Δ*vapB*Δ*vapK1-vapM* mutant. Deletion of the *vapK1-vapM* region was verified by PCR using primer pairs (S3 Table) annealing outside of the deletion site. As observed, DNA template from the mutant strain produced an expected 1.0 kb band due to the loss of the *vapK1-vapM* region (S2 Fig) in contrast to wild type 33705 DNA which yielded no amplicon (S2 Fig) because the region is too large to amplify under the PCR conditions used.

**Fig 3:**
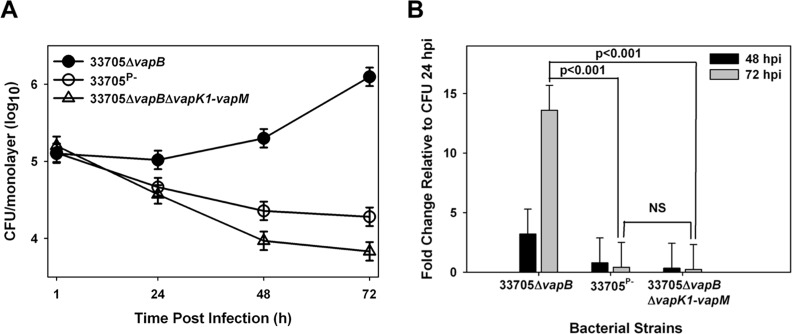
Deletion of *vapK1* through *vapM* of the PAI region of the pVAPB-type plasmid abolishes the intramacrophage replicative ability of the *R*. *equi* strain 33705. Murine BMDMs were infected at an MOI of 10:1 with the *R*. *equi* strains 33705Δ*vapB*, 33705^P-^, and 33705Δ*vapB*Δ*vapK1*-*vapM* and intracellular growth was followed by lysis and plating of triplicate macrophage monolayers over 72 h post infection (hpi) (A) and fold change in CFU of intracellular bacteria at 48 and 72 hpi relative to 24 hpi (B) was determined. Statistical analysis was performed on a compilation of 3 individual experiments. Error bars represent standard deviation from the mean.

Analysis of the Δ*vapB*Δ*vapK1-vapM* mutant in macrophages revealed it unable to replicate and in fact, its numbers decreased 10-fold over 72 hours, a growth profile similar to that of the plasmid-cured derivative used as a negative control for intracellular growth in this assay (Fig 3A and 3B). In contrast, the 33705*ΔvapB* strain (parent background) replicated ~15-fold during this time frame such that at 72 hpi, its intracellular numbers were greater than 2 logs higher than that of the Δ*vapB*Δv*apK1-vapM* mutant. These data demonstrate that the intracellular replication-required component(s) of the pVAPB-type plasmid lay within the *vapK1* to *vapM* region of the PAI.

### *vapK1* or *vapK2* is required for intramacrophage replication

**Fig 4:**
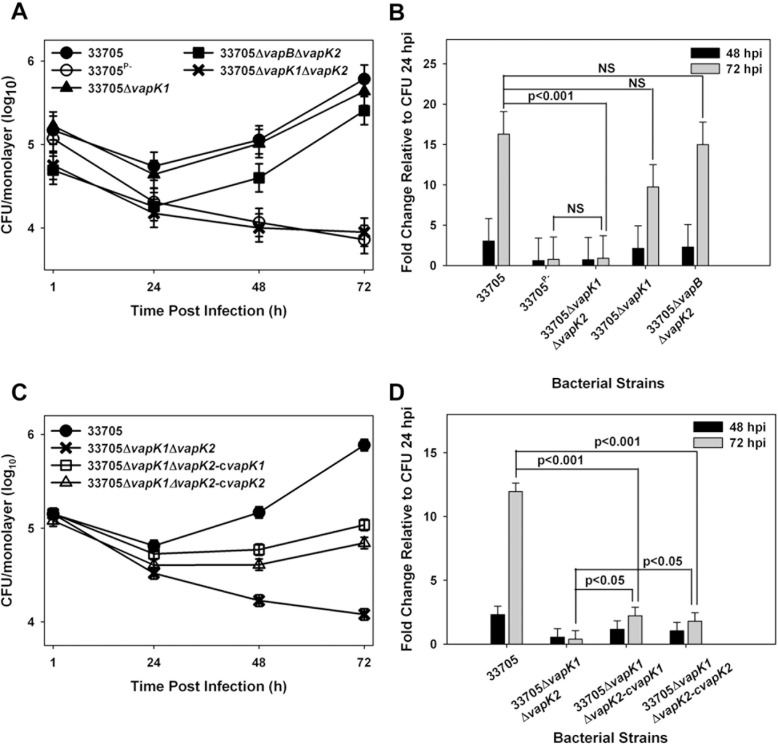
Deletion of *vapK1* and *vapK2* abolishes intramacrophage replication. Intracellular growth of *R*. *equi* strains 33705, 33705^P-^, 33705Δ*vapK1*, 33705Δ*vapB*Δ*vapK2*, and 33705Δ*vapK1*Δ*vapK2* was assessed by lysis and plating of triplicate bone marrow-derived macrophage monolayers over 72 h post infection (hpi) in BMDMs (A) and fold change of intracellular bacteria at 48 and 72 hpi relative to 24 hpi (B) is shown. Similarly, the intracellular growth of mutant strains expressing either *vapK1* (33705Δ*vapK1*Δ*vapK2*-*cvapK1*) or *vapK2* (33705Δ*vapK1*Δ*vapK2*-*cvapK2*) from the *Mycobacterium spp*. *hsp60* promoter in BMDMs was compared to wild type 33705 and the 33705Δ*vapK1*Δ*vapK2* mutant by lysis and plating of triplicate macrophage monolayers over 72 h post infection (hpi) (C) and fold change of intracellular bacteria at 48 and 72 hpi relative to 24 hpi (D) was determined. Statistical analysis was performed on a compilation of 3 individual experiments. Error bars represent standard deviation from the mean.

Interestingly, held within the *vapK1* to *vapM* region are *vapK1* and *vapK2*, two genes that are virtually identical to one another, suggesting functional redundancy that could potentially attribute to virulence. Aside from VapB, the predicted proteins encoded by *vapK1* and *vapK2* are the most highly related pVAPB Vap family members to the pVAPA-encoded VapA protein with 57% identity and 67% similarity [9]. Additionally, VapK1 and VapK2 share 99% identity and vary by only one amino acid, a glycine at residue 60 of VapK1 which is an aspartic acid in VapK2 [9]. Due to the observed redundancy and similarity to *vapA*, we hypothesized that one or both of the *vapK* genes could allow for intramacrophage replication of pVAPB-containing *R*. *equi* isolates. Therefore, three additional deletion mutants were created (Fig 2) and analyzed. First a marked, individual *vapK1* deletion mutant was made on a clean (unmarked) wild type background. Secondly, an unmarked, in-frame *vapK2* deletion was constructed on the Δ*vapB* mutant background. An in frame deletion was preferred here to avoid any potential alterations in gene expression of the neighboring *vapL* and *vapM* genes. The marked Δ*vapB* background was used because *R*. *equi* has a tendency to lose the virulence plasmid during the subculturing required for the allelic replacement process making it difficult to obtain an unmarked deletion unless antibiotic pressure is applied to ensure virulence plasmid retention. Lastly, an unmarked, inframe *vapK2* deletion was added to the individual, marked Δ*vapK1* mutant, resulting in a strain that possessed a pVAPB plasmid lacking both *vapK1* and *vapK2* only, with all other plasmid genes intact. Both the *vapK1* and *vapK2* deletions were verified using PCR analysis, where the loss of *vapK1* was confirmed using a forward primer that anneals internal to the zeocin resistance cassette and a reverse primer annealing external to the deletion site, resulting in a 1.2 kb pair amplicon (S3 Table; S3 Fig) in the absence of *vapK1*. In contrast, no product was produced using DNA from the wild type 33705 pVAPB containing *R*. *equi* isolate. The removal of *vapK2* was verified by PCR analysis using a forward primer that annealed inside of *vapL* and a reverse primer that annealed inside of *vapM*, resulting in an amplicon of ~1.5 kb when *vapK2* is present and a smaller one of 860 bp in the absence of *vapK2* (S3 Table; S4A Fig). In order to verify that the expression of *vapL* and *vapM* was not disrupted during the creation of the *vapK2* deletion, the expression of these two genes during mid-logarithmic stage growth were analyzed by qRT-PCR and demonstrated to be equivalent to that of wild type 33705 (S4B Fig). Next, the growth potential of these three mutant strains was examined in macrophages. The deletions of *vapK1* and *vapK2* individually did not compromise intramacrophage replication capacity and these mutants displayed a magnitude of intracellular growth at 72 hr post infection comparable to that of wild type 33705, and significantly higher than the plasmid cured 37705^P-^ strain (Fig 4A and 4B). In contrast, the Δv*apK1*Δ*vap*K2 double deletion mutant could not replicate intracellularly and showed an intramacrophage growth phenotype similar to that of the avirulent plasmid-cured derivative (Fig 4A and 4B). These data show that while deletion of an individual *vapK* gene is not detrimental, the absence of both *vapK1* and *vapK2* is fully attenuating for intracellular growth demonstrating the importance of these genes.

To further demonstrate the importance of *vapK1* and *vapK2* for intracellular replication, a complementation analysis was performed. The Δ*vapK1*Δ*vapK2* mutant of strain 33705 was transformed with an episomal plasmid (pMV261.hyg) with *vapK1* or *vapK2* individually expressed from the *Mycobacterium spp*. *hsp60* promoter. The complementation analysis demonstrated that while expression of *vapK1* or *vapK2* individually did not fully restore the capacity for intracellular replication to the Δ*vapK1*Δ*vapK2* deletion mutant, partial complementation was achieved (Fig 4C and 4D). To assess whether partial complementation was achieved because of insufficient gene expression in the complementing constructs, the expression levels of *vapK1* or *vapK2* from these constructs was assessed during an *in vitro* macrophage infection by qRT-PCR analysis. Due to the virtual identity of *vapK1* and *vapK2* (607 out of 609 base pairs identical), it was not possible to distinguish between expression of the two *vapK* genes in the wild type 33705 isolate, therefore, the expression of total *vapK* mRNA (*vapK1* and *vapK2*) was evaluated instead of individual *vapK1* or *vapK2* mRNA production. It was determined that total *vapK1/K2* expression from the pMV261.hyg expression vector was equivalent to that of the native *R*. *equi* promoters during macrophage infection (S5A Fig), however, differing protein levels might account for lack of full complementation in these experiments.

### The pVAPB-encoded VapK proteins are functionally equivalent to that of the pVAPA-encoded VapA

**Fig 5:**
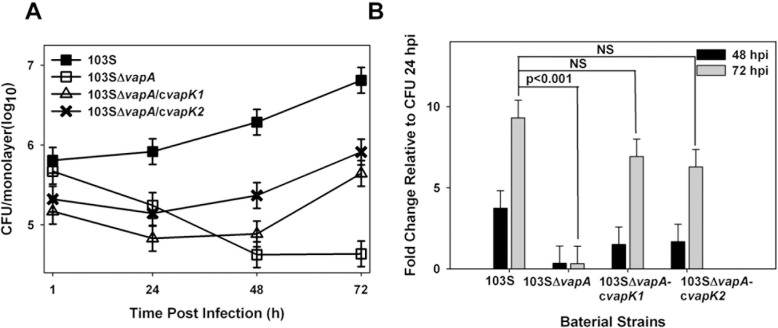
VapK is functionally equivalent to VapA. Bacterial growth of 103ΔvapA complemented with either *vapK1* or *vapK2* (strains 103Δ*vapA*/c*vapK* and 103ΔvapA/c*vapK2*, respectively) was followed and compared to wild type 103S carrying the pVAPA plasmid and 103Δ*vapA* by standard lysis and plating of triplicate BMDM monolayers over 72 h post infection (hpi) (A). Fold change of intracellular bacteria at 48 and 72 hpi relative to 24 hpi (B) was established. Data and statistical analysis were performed on a compilation of 2 individual experiments. Error bars represent standard deviation from the mean.

The necessity of the pVAPA-encoded *vapA* gene is well documented, and it is known that deletion of this gene results in loss of the ability to replicate in macrophages and virulence *in vivo* [14, 25–29]. In order to determine if the pVAPB-encoded VapK proteins are functionally equivalent to VapA, a complementation analysis was performed wherein an *R*. *equi* strain carrying pVAPA deleted of *vapA*, strain 103Δ*vapA*, was transformed with the same episomal plasmid expressing either *vapK1* or *vapK2*, used in the aforementioned complementation analysis of the Δ*vapK1*Δ*vapK2* double deletion mutant. As demonstrated in Fig 5A and 5B, expression of either *vapK1* or *vapK2* rescued the growth defect of the Δ*vapA* mutant and restored replicative ability to the strain showing that the VapK proteins can functionally substitute for VapA. The intracellular bacterial numbers of the of the *vapK* complementing strains (103SΔ*vapA*/c*vapK1* and 103SΔ*vapA*/c*vapK2*) at 72 hours post infection were not significantly different from wild type and were dramatically greater (~1.5-2logs higher) than the Δ*vapA* mutant, although there was a tendency toward a reduction in replicative ability of these complemented strains early in infection, as compared to wild type 103S carrying pVAPA. Despite the lag in intracellular growth, the mRNA levels of *vapK1* or *vapK2* were found to be equivalent at 24 hpi to the *vapK* expression under the native promoters found in pVAPB-carrying 33705 (S5B Fig). These data indicate that VapK encoded on the pVAPB-type plasmid is functionally similar to VapA produced by the pVAPA-type plasmid typically carried by equine *R*. *equi* isolates.

**Fig 6:**
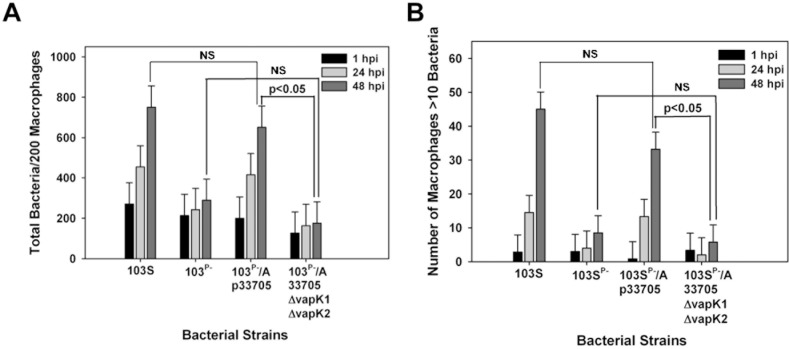
VapK is required for growth of a transconjugant *R*. *equi* strain in equine macrophages. Intracellular growth of *R*. *equi* strains 103S, 103^P-^, 103^P-^/A-p33705, 103^P-^/A-p33705Δ*vapK1*Δ*vapK2* was assessed in equine alveolar macrophages at an MOI of 5:1. Triplicate monolayers were fixed at 1h, 24h, and 48h post-infection, and stained as described in *Materials and Methods*. The number of bacteria per 200 macrophages (A) and the number of macrophages with greater than 10 bacteria per 200 macrophages (B) was enumerated under fluorescence microscopy. Error bars represent standard deviation from the mean. Data and statistical analysis were performed on a compilation of 2 individual experiments using macrophages obtained from two different equine donors.

Previous work of our laboratory demonstrated that conjugal transfer of the 33705 pVAPB-type plasmid to the equine-derived pVAPA plasmid-cured 103S^P-^ strain yielded a transconjugant (103S^P-^/A-p33705) that was able to replicate within murine and equine macrophages [20]. Those results indicated that the chromosome of an equine *R*. *equi* isolate is able to engage in crosstalk with the gene products of a non-native plasmid (pVAPB) obtained from an *R*. *equi* isolate from a different host species (swine) to create an appropriate intracellular niche suitable for bacterial replication. We hypothesized that the pVAPB plasmid containing deletions in *vapK1* and *vapK2* would not be able to support the growth of the equine plasmid-cured 103S^P-^ strain. To assess the latter, the Δ*vapK1*Δ*vapK2* mutant of 33705 was mated with strain 103S^P-^ and the intracellular growth capability of the resultant transconjugant (103S^P-^/A-p33705Δ*vapK1*Δ*vapK2*) was assessed in equine alveolar macrophages, a natural host cell for *R*. *equi*. It is important to note that *R*. *equi* growing in equine alveolar macrophages often forms chains and clusters of organisms within vacuoles which tend to clump when the monolayers are lysed. Therefore, traditional lysis and plating of infected macrophage monolayers has a tendancy to under represent the total number of bacteria present in the monolayer. Thus, in this cell type, bacterial replication over time is followed by immunostaining infected monolayers and using fluorescent microscopy to enumerate the number of bacteria per 200 macrophages in triplicate at each time point [12, 20, 24]. In parallel, as an additional assessment, the number of macrophages with 10 or greater bacteria is recorded as previously described [12, 20, 24]. As expected [20], the wild type equine isolate 103S carrying its native pVAPA-type plasmid and the transconjugant 103S^P-^/A-p33705 in possession of the intact non-native pVAPB-type plasmid increased in number in these macrophages over time (Fig 6). At 1h post-infection only approximetly 5 of 200 marophages possessed greater than 10 bacteria, whereas, 48h post-infection this number was 10 times greater. In contrast, the transconjugant 103S^P-^/A-p33705Δ*vapK1*Δ*vapK2* was unable to replicate within these macrophages displaying a phenotype much like that of the plasmid-cured 103S^P-^ strain. The latter finding provides additional support for the contention that VapK functions to promote conditions favorable for the replication of *R*. *equi* in host macrophages.

## Discussion

Although the pVAPA-, pVAPB- and pVAPN-type plasmids carried by disease causing strains of *R*. *equi* isolates differ greatly in their PAI regions [9, 10], there are conserved components, the most intriguing of which is the presence of multiple members of the unique and *R*. *equi* specific *vap* gene family, all of which share considerable homology at their C-termini [9, 10]. Amplification of *vap* genes is evident across all plasmid types perhaps indicative of selective pressures driving the maintenance of the *vap* genes in these plasmids. However, it is clear from this work and our previously published research [14, 24], that the *vap* genes are not equivalent in function.

All the Vap proteins share a highly conserved C-terminus, with a variable N-terminus [9]. The crystal structures of VapB, VapD, and VapG were determined after the removal of the unstable variable N-terminus and reveal an 8 strand ß-barrel domain along with a single peripheral α-helix, representing the conserved C-terminus [30, 31]. The protein fold is described as two Greek-key motifs separated by a small α-helix, a topology that has not been observed elsewhere and is a novel fold family [30–32]. While the topology of the Vap proteins is novel, there are partial similarities to other 8 strand ß-barrel proteins that are associated with bacterial virulence [31]. Some of the speculated functions of the Vap proteins are roles in bacterial adhesion and/or entry into mammalian cells or inhibition of host enzymes critical to antimicrobial defense [31]. The structure of the conserved ß-barrel domain has no significant depression or indentations that would suggest the presence of ligand binding or enzyme activity, however, this does not exclude the possibility that the Vaps are involved in interactions with other proteins or host lipids. In support of the latter, we have recently demonstrated that VapA can bind synthetic liposomes containing phosphatidic acid (PA) [16], a component of mammalian membranes. Additionally, it is feasible that the Vap proteins could undergo a conformational change in the presence of environmental cues, such as an increase in temperature, or change in the pH [30]. In fact, we noted that VapA:PA binding is enhanced at lower pH [16].

While little is known about the functionality of the Vap proteins, the best studied Vap family member is VapA, the key virulence determinant encoded by the pVAPA plasmid. The exact mechanism of action of this ~17kDa cell envelope-associated protein remains enigmatic [33] but it is essential for survival and intracellular replication in macrophages [14]. Interestingly, during macrophage infection, VapA can be found on both the bacterial surface and on the membrane of the *R*. *equi*-containing vacuole (RCV) [16]. We speculate that the lipid binding property of VapA serves to localize the protein to the RCV membrane wherein it is well-positioned to influence phagosome development. Consistent with this, we recently showed that later in infection, RCV displaying VapA at the membrane and containing replicating *R*. *equi* were lacking lysosomal membrane protein 1 (LAMP-1), whereas RCV without VapA at the membrane were LAMP-1 positive and contained fewer bacteria. These findings imply that membrane-associated VapA influences RCV interaction with lysosomal vesicles helping to thwart bacterial elimination. Ultimately, perturbation of RCV development allows the bacteria to replicate within the macrophage until the host cell undergoes necrosis resulting in the spread of the bacterium to surrounding macrophages [34]. Whether RCV generated in response to infection by pVAPB-containing *R*. *equi* follows a similar path is unknown but is of interest.

Studies of pVAPB-type plasmid-carrying *R*. *equi* strains are limited, however work of our laboratory has recently established that this plasmid type can confer intramacrophage replication capabilities [20]. The *vap* genes of the pVAPB-type plasmid typically carried by swine and many human *R*. *equi* isolates are distinct from those found on the pVAPA-type plasmid. Because of the necessity of *vapA*, we hypothesized that there is a component(s) found on the pVAPB-type plasmid that functions similarly to *vapA* and proposed that such a component would be a *vap* family member. Through the analysis of numerous deletion mutants, it was determined that deletion of both *vapK1* and *vapK2* results in a strain fully attenuated for intramacrophage growth. Interestingly, when these genes were deleted individually, intracellular replication was minimally impacted. *vapK1* and *vapK2* vary by only one amino acid, a glycine and an aspartic acid respectively. This is a prime example of bacterial functional redundancy, meaning that the product of each *vapK* gene can compensate in the event one of the genes is rendered non-functional. While gene duplication is a common occurrence in almost all bacterial species, duplicated genes are not thought to be fixed in the genome unless the selective pressure to maintain them is greater than the fitness cost required to keep them [35, 36]. Thus it is possible if differences in functionality exist between VapK1 and VapK2 such may come into play in another aspect of bacterial pathogenesis such as in interactions with other host cell types or in evasion of the host immune system.

Interestingly, the VapK proteins are not the *vap* gene protein products with the highest degree of amino acid sequence similarity to the pVAPA-encoded VapA protein. VapK1 and VapK2 share 57% identity and 67% similarity to VapA, whereas VapB has 75% identity and 81% similarity [9]. Therefore, it was surprising that deletion of *vapB* had no impact on the replicative ability of the pVAPB-type plasmid-containing *R*. *equi* isolate. However, when the 103Δ*vapA* strain was provided either *vapK1* or *vapK2*, the ability of the bacteria to replicate in murine macrophages was restored to near wild type levels. These findings are further supported by additional data produced in our laboratory showing that the exogenous addition of soluble VapK2 but not VapB protein to infected murine macrophage monolayers reversed the intramacrophage growth defect of the *ΔvapA* mutant [16]. Interestingly, in contrast to VapA, VapK2 does not bind phosphatidic acid [16], but it may well bind a different lipid component of mammalian membranes. Evaluating the lipid binding capacity of VapK is of particular interest as is determining the location of VapK during macrophage infection, but doing such is well beyond the scope of this work.

Though VapK is not the most highly related Vap protein to VapA, it is the most similar to VapN, the *vap* gene protein product critical for intramacrophage replication of *R*. *equi* isolates housing the pVAPN-type plasmid. These two proteins share 80% similarity and 72% identity. Similar to the VapK protein, VapN shares only 56% identity and 69% similarity to VapA, suggesting perhaps that VapK and VapN are more closely related evolutionally relative to VapA. It would be of interest to confirm whether the expression of *vapN* can complement the *vapK* null mutant and vice versa. While all three Vap proteins are crucial for bacterial intramacrophage growth, the differences in protein sequence may reflect evolutionary adaptation to specific host niches and purport specific or preferred host:ligand interactions. Determining such will be the focus of future experimentation.

## Supporting information

S1 Fig Deletion of *vapB* does not affect the intracellular replication capacity of the *R*. *equi* strain 33705 containing a pVAPB-type plasmid. The deletion of *vapB* was confirmed through PCR analysis using primer pairs (S3 Table) that anneal internal (A) and external (B) to the *vapB* gene. Results obtained for the *vapB* mutant are shown in the right most lanes labeled Δ. The second and third lanes are the products of control reactions using total genomic DNA from the wild type parent 33705 carrying a pVAPB-type plasmid or from its isogenic plasmid-cured derivative strain 33705^P-^as template, indicated by + and–symbols respectively. Standard molecular weight DNA markers (M) are in the left most lanes. Intracellular growth was determined by standard lysis and plating of murine BMDM in triplicate infected with *R*. *equi* strains 33705, 33705^P-^, and 33705Δ*vapB* using an MOI of 10:1. The intracellular growth was assessed over 72 h post infection (hpi) (C) and fold change in CFU of intracellular bacteria at 48 and 72 hpi relative to 24 hpi was determined (D). Statistical analysis was done on a compilation of 3 individual experiments. Error bars represent the standard deviation from the mean. NS: not significant.
(TIF)

S2 Fig PCR Confirmation of the Δ*vapK1-vapM* genotype. PCR analysis confirming the deletion of *vapK1-vapM* using a primer pair that anneals external to the deletion site. Results obtained for the Δ*vapB*Δ*vapK1-VapM* mutant are shown in the right most lanes labeled Δ. The second and third lanes are the products of control reactions using total genomic DNA from the 33705Δ*vapB* mutant or from its isogenic plasmid-cured derivative strain 33705^P-^as template, indicated by + and–symbols respectively. Standard molecular weight DNA markers (M) are in the left most lane.
(TIF)

S3 Fig PCR Confirmation of the Δ*vapK1* genotype. PCR analysis confirming the deletion of *vapK1* using a primer pair wherein the forward primer anneals internal to the zeocin cassette marking the mutation site and the reverse primer anneals external to the *vapK1* deletion site. The amplicon produced with template from Δ*vapK1* mutant is shown in the right most lane labeled Δ. The second and third lanes are the products of control reactions using total genomic DNA from strain 33705 carrying a pVAPB-type plasmid or from plasmid-free strain 33705^P-^as template, indicated by + and–symbols respectively. Molecular weight DNA standards (M) are in the far left lane.
(TIF)

S4 Fig Confirmation of the Δ*vapK2* genotype. Deletion of *vapK2* (shown in lane 4 labeled Δ) was confirmed by PCR analysis (A). Amplicon produced using template from wild type strain 33705 is shown in lane 2 (+) and from its pVAPB-type plasmid-cured derivative strain 33705^P-^ is in lane 3 (-). Molecular DNA markers (M) are shown in lane 1. Expression of *vapL* and *vapM* in the Δ*vapK2* mutant using qRT-PCR analysis as described in *Materials and Methods* (B).
(TIF)

S5 Fig Expression of *vapK* complemented strains during macrophage infection. *vapK* mRNA expression levels of *R*. *equi* strains 33705Δ*vapK*1Δv*apK2* (A) and 103Δ*vapA* (B) complemented with either *vapK1* or *vapK2* during *in vitro* macrophage infection using qRT-PCR analysis as described *Materials and Methods*.
(TIF)

S1 Table Plasmids used in this study. (DOCX)

S2 Table Bacterial strains used in this study. (DOCX)

S3 Table Oligonucleotides used in this study. (DOCX)
