# Supplementary figures and images for: Identification of a VapA virulence factor functional homolog in Rhodococcus equi isolates housing the pVAPB plasmid
Source: PLoS One. 2018 Oct 4;13(10):e0204475. doi: 10.1371/journal.pone.0204475 (PMC6171844; doi:10.1371/journal.pone.0204475)

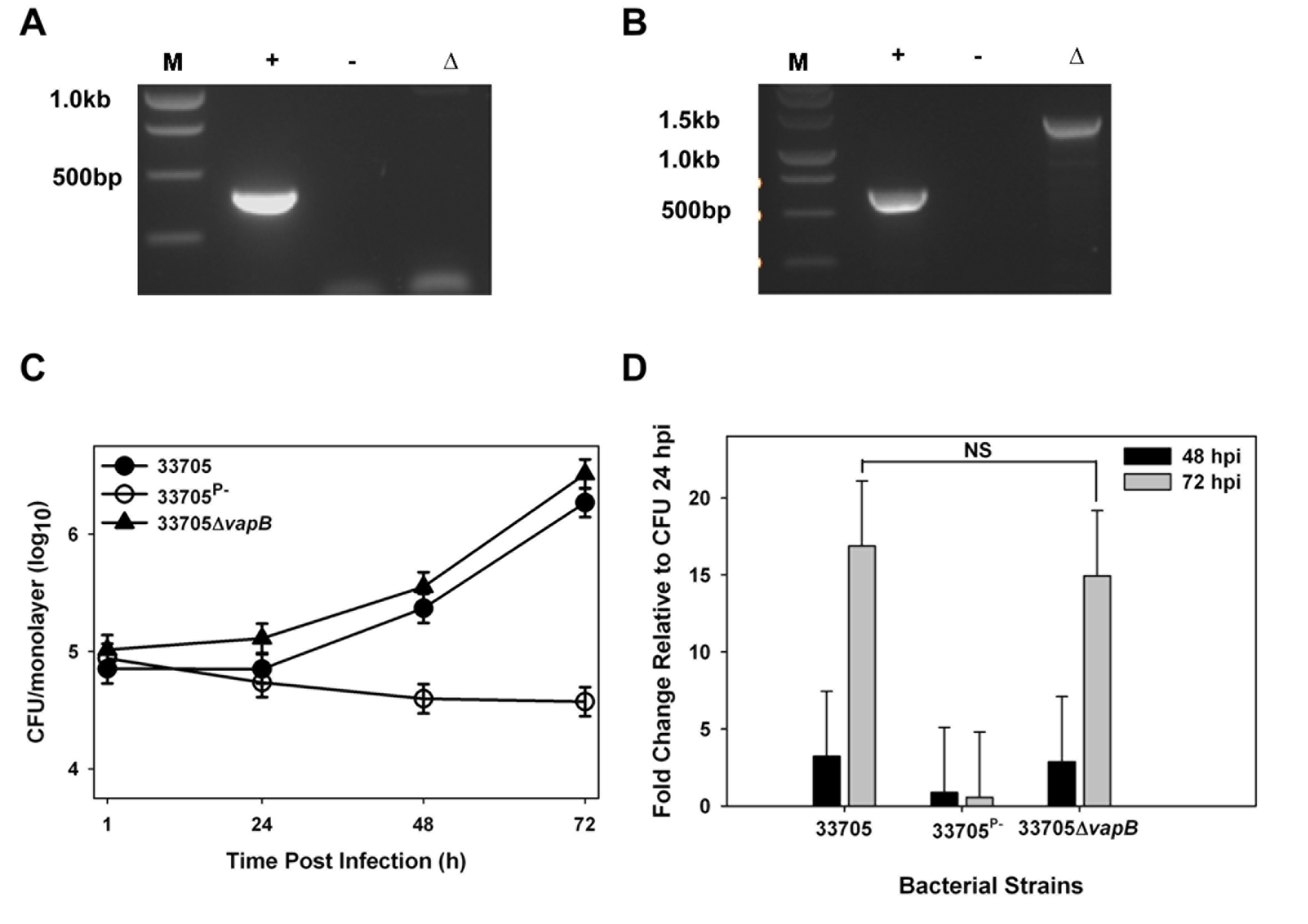

Supplement: S1 Fig — The deletion of vapB was confirmed through PCR analysis using primer pairs (S3 Table) that anneal internal (A) and external (B) to the vapB gene. Results obtained for the vapB mutant are shown in the right most lanes labeled Δ. The second and third lanes are the products of control reactions using total genomic DNA from the wild type parent 33705 carrying a pVAPB-type plasmid or from its isogenic plasmid-cured derivative strain 33705P-as template, indicated by + and–symbols respectively. Standard molecular weight DNA markers (M) are in the left most lanes. Intracellular growth was determined by standard lysis and plating of murine BMDM in triplicate infected with R. equi strains 33705, 33705P-, and 33705ΔvapB using an MOI of 10:1. The intracellular growth was assessed over 72 h post infection (hpi) (C) and fold change in CFU of intracellular bacteria at 48 and 72 hpi relative to 24 hpi was determined (D). Statistical analysis was done on a compilation of 3 individual experiments. Error bars represent the standard deviation from the mean. NS: not significant. (TIF) [file pone.0204475.s001.tif]

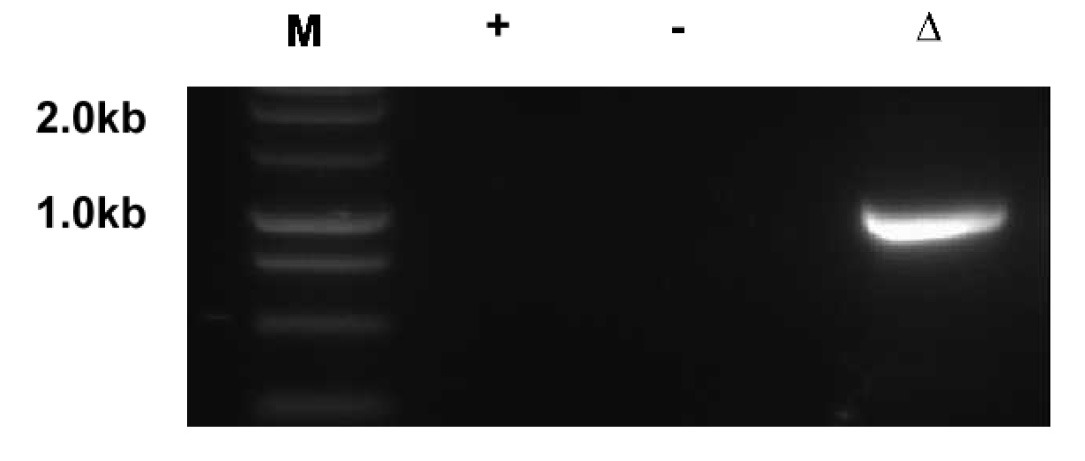

Supplement: S2 Fig — PCR analysis confirming the deletion of vapK1-vapM using a primer pair that anneals external to the deletion site. Results obtained for the ΔvapBΔvapK1-VapM mutant are shown in the right most lanes labeled Δ. The second and third lanes are the products of control reactions using total genomic DNA from the 33705ΔvapB mutant or from its isogenic plasmid-cured derivative strain 33705P-as template, indicated by + and–symbols respectively. Standard molecular weight DNA markers (M) are in the left most lane. (TIF) [file pone.0204475.s002.tif]

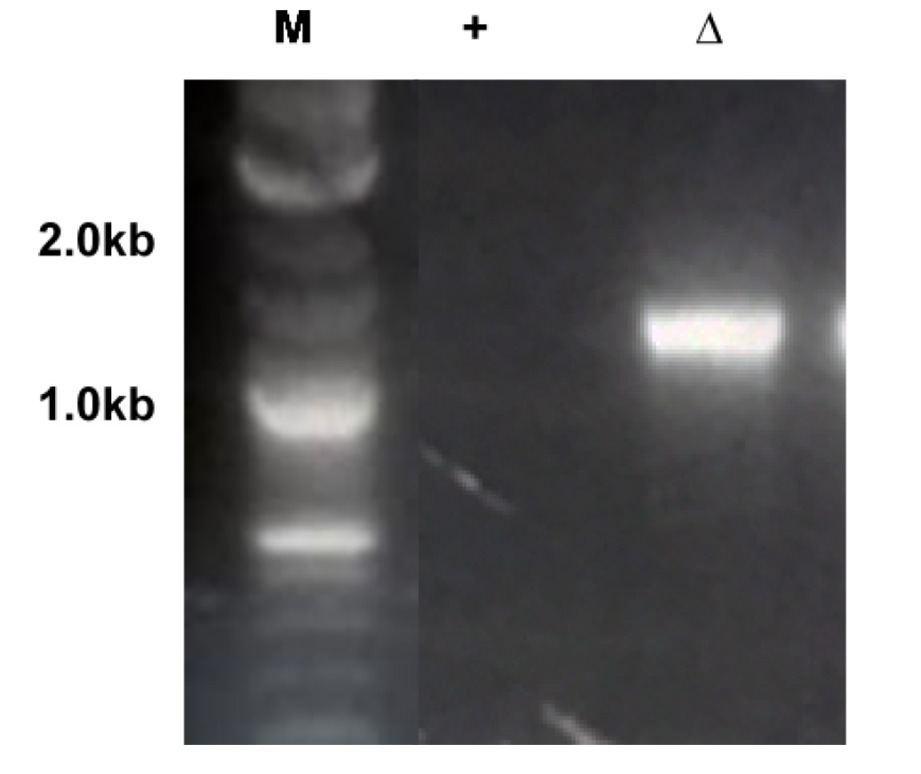

Supplement: S3 Fig — PCR analysis confirming the deletion of vapK1 using a primer pair wherein the forward primer anneals internal to the zeocin cassette marking the mutation site and the reverse primer anneals external to the vapK1 deletion site. The amplicon produced with template from ΔvapK1 mutant is shown in the right most lane labeled Δ. The second and third lanes are the products of control reactions using total genomic DNA from strain 33705 carrying a pVAPB-type plasmid or from plasmid-free strain 33705P-as template, indicated by + and–symbols respectively. Molecular weight DNA standards (M) are in the far left lane. (TIF) [file pone.0204475.s003.tif]

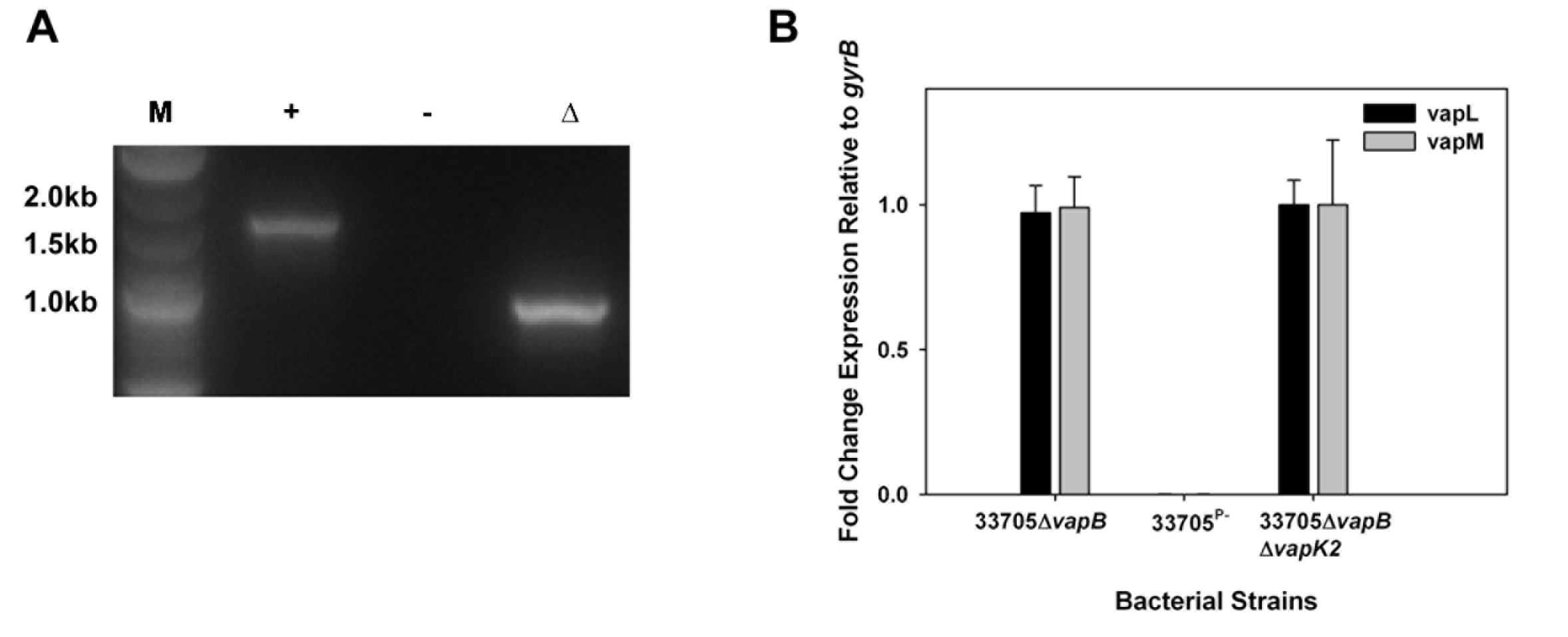

Supplement: S4 Fig — Deletion of vapK2 (shown in lane 4 labeled Δ) was confirmed by PCR analysis (A). Amplicon produced using template from wild type strain 33705 is shown in lane 2 (+) and from its pVAPB-type plasmid-cured derivative strain 33705P- is in lane 3 (-). Molecular DNA markers (M) are shown in lane 1. Expression of vapL and vapM in the ΔvapK2 mutant using qRT-PCR analysis as described in Materials and Methods (B). (TIF) [file pone.0204475.s004.tif]

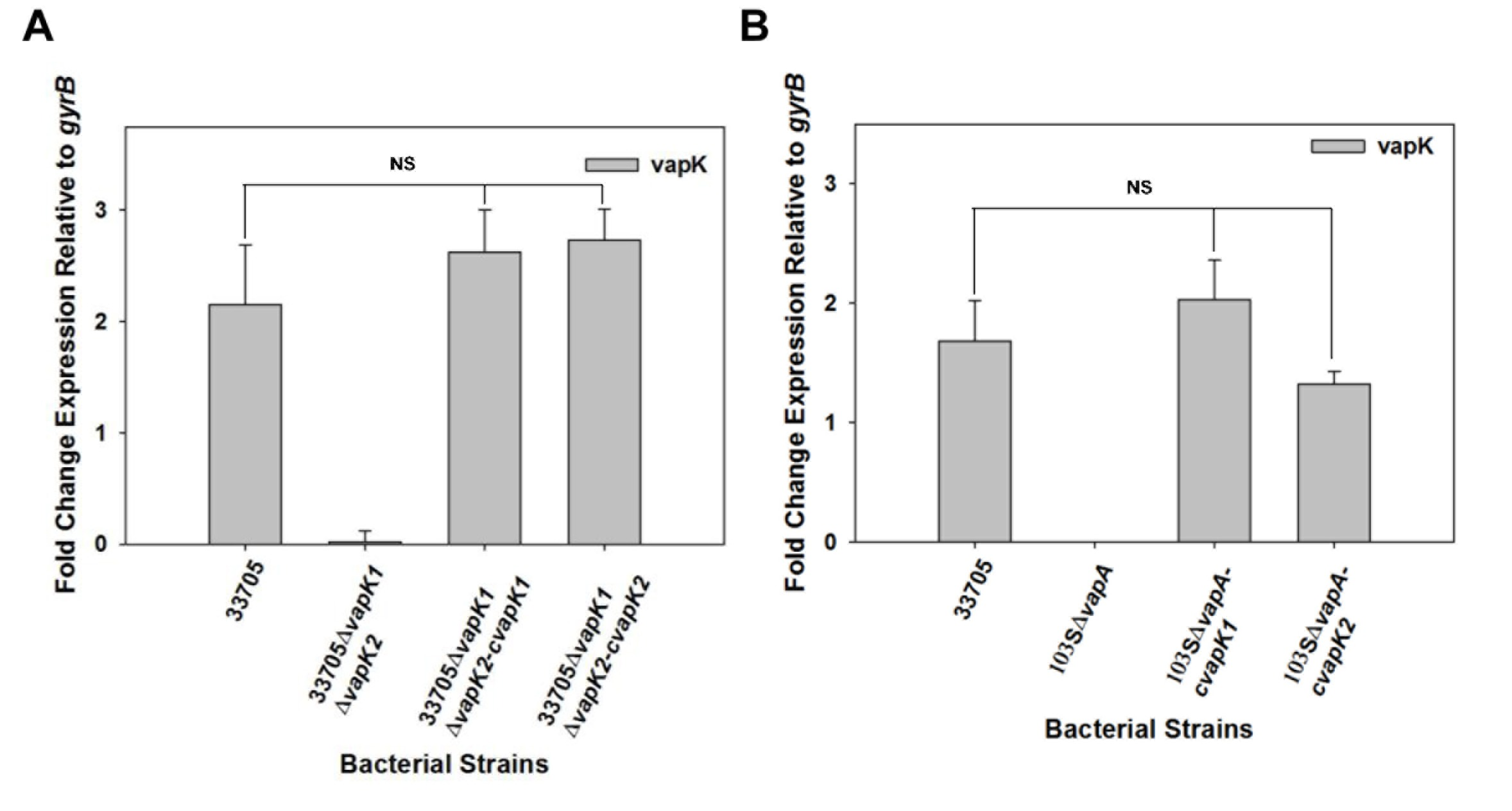

Supplement: S5 Fig — vapK mRNA expression levels of R. equi strains 33705ΔvapK1ΔvapK2 (A) and 103ΔvapA (B) complemented with either vapK1 or vapK2 during in vitro macrophage infection using qRT-PCR analysis as described Materials and Methods. (TIF) [file pone.0204475.s005.tif]
