# Supplementary material for: Identification of a VapA virulence factor functional homolog in Rhodococcus equi isolates housing the pVAPB plasmid
Source: PLoS One. 2018 Oct 4;13(10):e0204475. doi: 10.1371/journal.pone.0204475 (PMC6171844; doi:10.1371/journal.pone.0204475)
Supplement: S1 Table — (DOCX) [file pone.0204475.s006.docx]

**S1 Table: Plasmids used in this study.**

| **Plasmid** | **Genotype or characteristics** | **Source** |
| --- | --- | --- |
| ***Plasmids*** |  |  |
| pSelAct | *lacZ*, *codA:upp,* Apr^R^ | [1] |
| pEM7/Zeo | pEM7 vector containing zeocin cassette under the control of EM-7 bacterial promoter, Zeo^R^ | ThermoFisher Scientific |
| pSET152 | Vector containing *[aac(3)-IV]*; phiC31 integrase, *attP*, Apr^R^ | [2] |
| pMV261.hyg | *Mycobacterium-E. coli* shuttle plasmid with *oriM*, *hsp60* promoter, Hyg^R^, | [3] |
| pMV261.hyg.vapK1 | pMV261.hyg *Mycobacterium- E. coli* shuttle episomal vector expressing *vapK1* from the *hsp60* promoter of *Mycobacterium spp*.; Hyg^R^ | this study |
| pMV261.hyg.vapK2 | pMV261.hyg *Mycobacterium- E. coli* shuttle episomal vector expressing *vapK2* from the *hsp60* promoter of *Mycobacterium spp*.; Hyg^R^ | this study |
| pJWL1.1 | pSelAct containing 962 bp upstream of pVAPB PAI region; *lacZ*, *codA::upp*, Apr^R^ | this study |
| pJWL1.2 | pJWL1.1 containing 935 bp downstream region of pVAPB PAI region; *lacZ*, *codA::upp,* Apr^R^ | this study |
| pJWL1.0.hyg | pJWL1.2 containing 1.3 kb hygromycin cassette inserted between the upstream and downstream PAI regions; *lacZ*, *codA::upp,* Apr^R^, Hyg^R^ | this study |
| pGB26 | pSelAct containing 848 bp upstream region of *vapB*; *lacZ*, *codA::upp*, Apr^R^ | this study |
| pGB26.1 | pGB26 containing the 770 bp downstream region of *vapB*; *lacZ*, *codA::upp*, Apr^R^ | this study |
| pGBC26hyg.1 | pSelAct containing 848 bp upstream region and 770 bp downstream region of *vapB* flanking a hygromycin resistance cassette; *lacZ*, *codA::upp*, Apr^R^, Hyg^R^ | this study |
| pJWL2.1 | pSelAct containing 938 bp upstream region of *vapK1;* *lacZ*, *codA::upp*, Apr^R^ | this study |
| pJWL2.0 | pJWL2.1 containing 747 bp downstream region of *vapM*; *lacZ*, *codA::upp*, Apr^R^ | this study |
| pJWL4.1 | pJWL2.1 containing 1.3 kb downstream *vapK1* region; *lacZ*, *codA::upp*, Apr^R^ | this study |
| pJWL4.0.zeo | pJWL4.2 with *vapK1* upstream and downstream regions flanking zeocin resistance cassette; *lacZ*, *codA::upp*, Apr^R^, Zeo^R^ | this study |
| pJWL5.1 | pSelAct vector containing 967 bp upstream region of *vapK2*; *lacZ*, *codA::upp*, Apr^R^ | this study |
| pJWL5.0 | pJWL5.1 containing 1.1 kb downstream region of *vapK2*; *lacZ*, *codA::upp*, Apr^R^ | this study |

1. van der Geize R, de Jong W, Hessels GI, Grommen AW, Jacobs AA, Dijkhuizen L. A novel method to generate unmarked gene deletions in the intracellular pathogen Rhodococcus equi using 5-fluorocytosine conditional lethality. Nucleic Acids Res. 2008;36(22):e151.

2. Hong Y, Hondalus MK. Site-specific integration of Streptomyces PhiC31 integrase-based vectors in the chromosome of Rhodococcus equi. FEMS Microbiol Lett. 2008;287(1):63-8.

3. Stover CK, de la Cruz VF, Fuerst TR, Burlein JE, Benson LA, Bennett LT, et al. New use of BCG for recombinant vaccines. Nature. 1991;351(6326):456-60.
