# Supplementary material for: Identification of a VapA virulence factor functional homolog in Rhodococcus equi isolates housing the pVAPB plasmid
Source: PLoS One. 2018 Oct 4;13(10):e0204475. doi: 10.1371/journal.pone.0204475 (PMC6171844; doi:10.1371/journal.pone.0204475)
Supplement: S2 Table — (DOCX) [file pone.0204475.s007.docx]

**S2 Table: Bacterial strains used in this study.**

| **Strain** | **Genotype or characteristics** | **Source** |
| --- | --- | --- |
| ***Escherichia coli*** |  |  |
| DH5α | F- Φ80lacZ∆M15, ∆(*lacZYA*-*argF*), U169 *recA1*, *endA1*, *hsdR17* (r_K_ ^-^ , m_K_ ^+^ ) *phoA* *supE*44 λ- *thi*-1 *gyrA*96 *relA1* | Zymo Research |
| ***Rhodococcus equi*** |  |  |
| 33705 | *R. equi* strain with virulence plasmid pVAPB originally isolated from the lymph node of a pig | ATCC |
| 33705^P-^ | Plasmid-cured variant of 33705 | [1] |
| 33705ΔPAI | 33705 deleted of the pVAPB PAI region; Hyg^R^ | this study |
| 33705Δ*vapB* | 33705 pVAPB plasmid deleted of *vapB*; Hyg^R^ | this study |
| 33705Δ*vapB* Δ*vapK1-vapM* | 33705Δ*vapB* containing a deletion of the *vapK1-vapM* region of the pVAPB plasmid; Hyg^R^ | this study |
| 33705Δ*vapK1* | 33705 containing a deletion of *vapK1* on the pVAPB plasmid; Zeo^R^ | this study |
| 33705Δ*vapB* Δ*vapK2* | 33705Δ*vapB* possessing a deletion of *vapK2*; Hyg^R^ | this study |
| 33705Δ*vapK1*Δ*vapK2* | 33705Δ*vapK1* containing a deletion of *vapK2*; Zeo^R^ | this study |
| 33705Δ*vapK1*Δ*vapK2*/c*vapK1* | 33705Δ*vapK1*Δ*vapK2* complemented with pMV261.hyg expressing *vapK1* from the *hsp60* promoter of *Mycobacterium spp*.; Zeo^R^, Hyg^R^ | this study |
| 33705Δ*vapK1*Δ*vapK2*/c*vapK2* | 33705Δ*vapK1*Δ*vapK2* complemented with pMV261.hyg expressing *vapK2* from the *hsp60* promoter from *Mycobacterium spp*.; Zeo^R^, Hyg^R^ | this study |
| 103S | Wild type strain with virulence plasmid pVAPA1037, expressing *vapA*; originally isolated from a pneumonic foal | [2] |
| 103S^P-^/A | 103S^P-^ containing a *[aac(3)-IV]* gene integrated on the chromosome; Apr^R^ | [1] |
| 103SΔ*vapA* | 103S containing an acc(3)-IV marked deletion of *vapA*; Apr^R^ | [3] |
| 103SΔ*vapA*/c*vapK1* | 103SΔ*vapA* complemented with pMV261.hyg expressing *vapK1* from the *hsp60* promoter of *Mycobacterium spp*.; Apr^R^, Hyg^R^ | this study |
| 103SΔ*vapA*/c*vapK2* | 103SΔ*vapA* complemented with pMV261.hyg expressing *vapK2* from the *hsp60* promoter of *Mycobacterium spp*.; Apr^R^, Hyg^R^ | this study |
| 103S^P-^/A-p33705 | Transconjugant of strain 103S^P-^/A and strain 33705*.*  It carries the pVAPB-type plasmid from 33705; Apr^R^ | this study |
| 103S^P-^/A-p33705Δ*vapK1*Δ*vapK2* | Transconjugant of strain 103^P-^/A and strain 33705Δ*vapK1*Δ*vapK2.*  It carries the pVAPB-type plasmid from 33705 deleted for *vapK1* and *vapK2*; Apr^R^, Zeo^R^ | this study |

1. Tripathi VN, Harding WC, Willingham-Lane JM, Hondalus MK. Conjugal transfer of a virulence plasmid in the opportunistic intracellular actinomycete Rhodococcus equi. J Bacteriol. 2012;194(24):6790-801.

2. Giguere S, Hondalus MK, Yager JA, Darrah P, Mosser DM, Prescott JF. Role of the 85-kilobase plasmid and plasmid-encoded virulence-associated protein A in intracellular survival and virulence of Rhodococcus equi. Infect Immun. 1999;67(7):3548-57.

3. Jain S, Bloom BR, Hondalus MK. Deletion of vapA encoding Virulence Associated Protein A attenuates the intracellular actinomycete Rhodococcus equi. Mol Microbiol. 2003;50(1):115-28.
