# Supplementary material for: Identification of a VapA virulence factor functional homolog in Rhodococcus equi isolates housing the pVAPB plasmid
Source: PLoS One. 2018 Oct 4;13(10):e0204475. doi: 10.1371/journal.pone.0204475 (PMC6171844; doi:10.1371/journal.pone.0204475)
Supplement: S3 Table — (DOCX) [file pone.0204475.s008.docx]

**S3 Table. Oligonucleotides used in this study.**

| **Name** | **Sequence (5’-3’)** |
| --- | --- |
| PAI-up (DraIII)-F | ATCACTACGTGTTCACCATCGCCTGCACTTC |
| PAI-up (SpeI)-R | CGTACTAGTGGACGGAGGTCCATCCTATAAC |
| PAI-down (SpeI)-F | GCTACTAGTTCGACGAGACACCGATAG |
| PAI-down (EcoRI)-R | CGGAATTCTCGGATGACGGGATAACG |
| PAI deletion-F | TCGACGACTTCGATGGTGAG |
| PAI deletion-R | TGTCGAGTGGTCCTTGAAAC |
| vapB-up-F (DraIII) | AATACACTACGTGGGCGCAAGGAAAG |
| vapB-up-R (SpeI) | AGTCACTAGTATCGCCCTAGACACC |
| vapB-down-F (SpeI) | AATAACTAGTGCGGGTCTGGAAGC |
| vapB-down-R (XmaI) | ACTTCCCGGGTTTCCGCACAATATG |
| vapB-int-F | GGCAGTGCCCTTCTTAAGGATG |
| vapB-int-R | AACTGCAGGGGCCTGGATATGG |
| vapB-ext-F | GAAGATCCGCGAAGGTTAAG |
| vapB-ext-R | TCAGCCTGCTATGCGTTATG |
| vapK1-up (DraIII)-F | GTCACTACGTGGTGACGAACAGAGATTCC |
| vapK1-up (SpeI)-R | GCGACTAGTGAAAGCGTCATTCGCTAC |
| vapK1-down (SpeI)-F | ATGCACTAGTTTGATGGGAACGGCGTACTG |
| vapK1-down (XmaI)-R | ATTACCCGGGAGCGGGTCTTCCATGACAAC |
| vapK1 deletion (zeo)-F | ACTTCGTGGAGGACGACTTC |
| vapK1 deletion-R | GCTGATCGGCAACACATACC |
| vakK2-up-F | /5Phos/GAGACGGTGTCGTTATACAG |
| vapK2-up-R | /5Phos/CGAGCAACGCGGGCAGATTCATAC |
| vapK2-down-F | /5Phos/GAAACCGGCAGCTGGTCATAAG |
| vapK2-down-R | /5Phos/GCATTCGATGGTGCAGGACTC |
| vapK2 deletion-F | GCGCAAAGCCGATGCAATAG |
| vapK2 deletion-R | TGCGTAAGCAGCACCTGTAG |
| vapK complement-F | /5Phos/TAGGGAACGCGCGCAGGTCCTG |
| vapK1 complement-R | /5Phos/CTAATCGAGCCTGTGACGTA |
| vapK2 complement-R | /5Phos/CAGTCTCGCTCGTTAAGCAG |
| REVP1 | GGAAGGAATGGCAAGAAA |
| REVP1c | TGTGCCGCTTCAAAGGCT |
| REVP4 | GACCTGTTCATAGCCGAG |
| REVP4c | TCGTCCTCGATCCGCTGC |
| REVP6 | GAGAGTTCAGTTTCGCGG |
| REVP6c | CCTTTCCATTGGTGTCTTC |
| REtrbA1 | GCGTCAGTGCGACAGTGATG |
| REtrbA1c | TCGGAGTCAGGTCGGAGG |
| 0440-F | CAGAGTTCTTCGACGACTTC |
| 0440-R | CGTAGGCTTCAACTACTTCC |
| vapL-F | CGACCGCACTCTCTATTACC |
| vapL-R | CCCTCTCGGAGGCTGTTATC |
| vapM-F | CTATGAGGAAGGACGGGAAG |
| vapM-R | CGACGGAGATTTCCTATACG |
| 0700-F | GCATAGCAGGCTGAAGGTAG |
| 0700-R | TGTCGAGTGGTCCTTGAAAC |
| gyr-qRT-F | GTCGAGCAGGGTCACGTGTA |
| gyr-qRT-R | AGCTCCTTTGCGTTCATCT |
| vapL-qRT-F | GTCGTCTTCGACGGTCAGTG |
| vapL-qRT-R | GCATCCAACGCGGCAACTTC |
| vapK-qRT-F | AGGTCCTGGGTGAAG |
| vapK-qRT-R | TGCTGCTGCCGTAAC |
| vapM-qRT-F | ATCAGCACCTGGAGATTACG |
| vapM-qRT-R | ACTCCCGCCGCATTAAATTC |

*Restriction enzymes are indicated by the underlined nucleotide sequence*
